# Underrecognized suicidality in higher functioning youth with autism spectrum disorder and attention deficit hyperactivity disorder

**DOI:** 10.1007/s44192-026-00453-0

**Published:** 2026-08-10

**Authors:** Mayank Gupta, James McCall, Priyal Khurana, Nihit Gupta

**Affiliations:** 1 Rutgers University, New Brunswick, NJ USA; 2 Independent Researcher, Boise, ID USA; 3 Mahatma Jyotirao Phoole University, Jaipur, India; 4 Department of Psychiatry, Dayton’s Children Hospital, Dayton, OH USA

## Abstract

**Objective:**

To examine factors associated with elevated risk of suicidal thoughts, behaviors, and death by suicide among high functioning individuals with autism spectrum disorder and attention deficit hyperactivity disorder, with particular emphasis on phenotypes characterized by high cognitive ability and delayed recognition.

**Methods:**

A structured review of the literature was conducted across multiple electronic databases using controlled vocabulary and key terms including suicidal risk, autism spectrum disorder, attention deficit hyperactivity disorder, high functioning, and neurodivergence. After screening for relevance, duplication, and methodological rigor, 7 studies meeting inclusion criteria were synthesized from an initial yield of fifty publications.

**Results:**

Across studies, individuals with ASD demonstrated a consistently elevated risk of STB and death by suicide compared with the general population, both over the lifetime and within shorter follow up periods. Risk was disproportionately concentrated among individuals without intellectual disability, with higher cognitive ability emerging as a vulnerability factor rather than a protective one. Communication differences, alexithymia, and impaired interoceptive awareness limited the sensitivity of caregiver and clinician assessments, contributing to under recognition of suicidality. Traditional protective factors such as educational attainment, employment, and relationship status did not reliably mitigate risk in ASD and ADHD populations. Camouflaging behaviors and diagnostic overshadowing frequently delayed identification, increasing exposure to cumulative psychosocial stress and untreated mental health conditions.

**Conclusions:**

High functioning individuals with ASD and co-occurring ADHD represent a concealed yet high risk population for suicidality. These findings underscore the urgent need for autism informed suicide risk assessment, early identification of neurodevelopmental traits in suicidal individuals, and prevention strategies tailored to cognitive profile, developmental history, and comorbidity. Advances in the current screening and intervention frameworks may mitigate adverse outcomes in this vulnerable group.

## Introduction

In the last decade there has been some critical development in understanding of autism spectrum disorder (ASD) subtypes and its association with a heightened risk of suicidal thoughts and behaviors (STB) [1]. Litman et al. used a generative mixture-modeling approach in a large, genetically characterized cohort to identify reproducible, clinically meaningful autism subtypes distinguished by patterns of core features, associated traits, and co-occurring conditions. These phenotypic classes mapped onto distinct genetic architectures and molecular pathways involving common, inherited, and de novo variants. Notably, differences in the developmental timing of affected genes aligned with variations in clinical outcomes, linking ASD heterogeneity to specific genetic programs and neurodevelopmental trajectories [2].

These developments provided much-needed insight, as historically, ASD was conceptualized mainly in terms of language impairment and intellectual disability, leading to underdiagnosis of individuals with average or above-average cognitive ability. As a result, individuals with normative or superior verbal and cognitive skills have often been overlooked or misdiagnosed. Diagnostic overshadowing and compensatory behaviors, such as social camouflage, further obscure clinical presentations and delay access to appropriate support [3–5].

Of particular interest is the Social and Behavioral Challenges subgroup, comprising approximately 37% of the cohort. Individuals in this class display social communication difficulties and restricted and repetitive behaviors while demonstrating relatively typical attainment of early developmental milestones [2]. This subgroup is further characterized by a high prevalence of co-occurring psychiatric conditions, including attention-deficit/hyperactivity disorder, anxiety, depressive disorders, and obsessive–compulsive disorder [6].

Within the heterogeneous autism spectrum, converging evidence indicates that individuals with relatively high cognitive ability and co-occurring neurodevelopmental conditions particularly attention-deficit/hyperactivity disorder (ADHD) constitute a subgroup at disproportionately elevated risk for STB [7]. These clinical presentations are often diagnostically complex, with overlapping symptom profiles that may obscure recognition of risk despite significant functional and affective burden.

Large registry-based studies have shown that co-occurring ADHD among individuals with ASD markedly increases the likelihood of intentional self-harm and suicide-related outcomes beyond ASD alone [8]. Importantly, the concurrent diagnosis of ASD and ADHD was not formally recognized until the DSM-5 in 2013, limiting earlier research of shared vulnerabilities and delaying systematic study of this high-risk phenotype [9]. Since this diagnostic revision, accumulating evidence indicates that individuals with co-occurring ASD and ADHD, particularly those without intellectual disability, are at substantially higher risk of suicide attempts and mortality than neurotypical peers. Meta-analytic estimates suggest that approximately one-third of individuals with ASD report lifetime suicidal ideation, and more than 15% report prior suicide attempts, underscoring the urgency of improved identification and prevention strategies [10].

A critical barrier to suicide prevention in neurodivergent populations is the limited validity of standard suicide risk assessments, which were developed for neurotypical individuals and often fail to capture atypical or internalized expressions of distress. This limitation is especially pronounced among cognitively able autistic individuals, women, and gender-diverse people, contributing to systematic under recognition of STB. Although autism-adapted measures for adults show promise, specific instruments such as the Suicidal Behaviors Questionnaire for Autism Spectrum Conditions (SBQ-ASC) and modified measures of suicidal ideation attributes (SIDAS-M) [11]. Validated screening tools for autistic children and adolescents particularly those with co-occurring ADHD remain scarce.

The phenomenology and pathways to STB in ASD differ from traditional models centered on acute stressors or attachment-based frameworks. Chronic social exclusion, persistent communication difficulties, repeated experiences of marginalization, and emotion regulation challenges interact with ADHD-related impulsivity and attentional dysregulation, potentially amplifying vulnerability to STB through cumulative developmental burden.

Against this background, the review aims to synthesize emerging evidence to clarify why cognitively able individuals with ASD and co-occurring ADHD represent a particularly high-risk subgroup for suicidal thoughts and behaviors. By integrating epidemiologic, clinical, and mechanistic perspectives, this review seeks to inform improved screening, earlier identification, and targeted interventions across mental health, educational, and community-based systems of care.

## Methods

A comprehensive search was conducted across various databases, including PubMed, Google Scholar, Scopus, Medline and Cochrane Library, from the inception to March 30, 2025. The inclusion criteria for this review were designed to encompass any published material focusing on suicidal risk vulnerability and assessment across all age groups, with a particular emphasis on neurodivergent. We intentionally kept our criteria broad to ensure that the review captured a wide range of studies, including those on children and adolescents. The search strategy employed controlled vocabulary and keywords such as “suicidal risk,” “high-functioning,” “autism spectrum disorder,” “attention-deficit hyperactivity disorder,” and “neurodivergent,” and was conducted in all languages. A manual search was also performed. To capture the most relevant results, the search included publications from the last 10 years, so as to also give an insight on gaps and future directions. The ultimate goal was to accumulate and analyze studies that provide an inner view of reality to guide efforts in identifying and providing next steps to be used in clinical settings. This search yielded 50 articles after removing duplicates. Following a review of the abstracts, 6 studies were deemed suitable for the initial draft of this narrative review, as agreed upon by the authors. Additionally, 4 other articles were included to support the narrative.

**Fig. 1:**
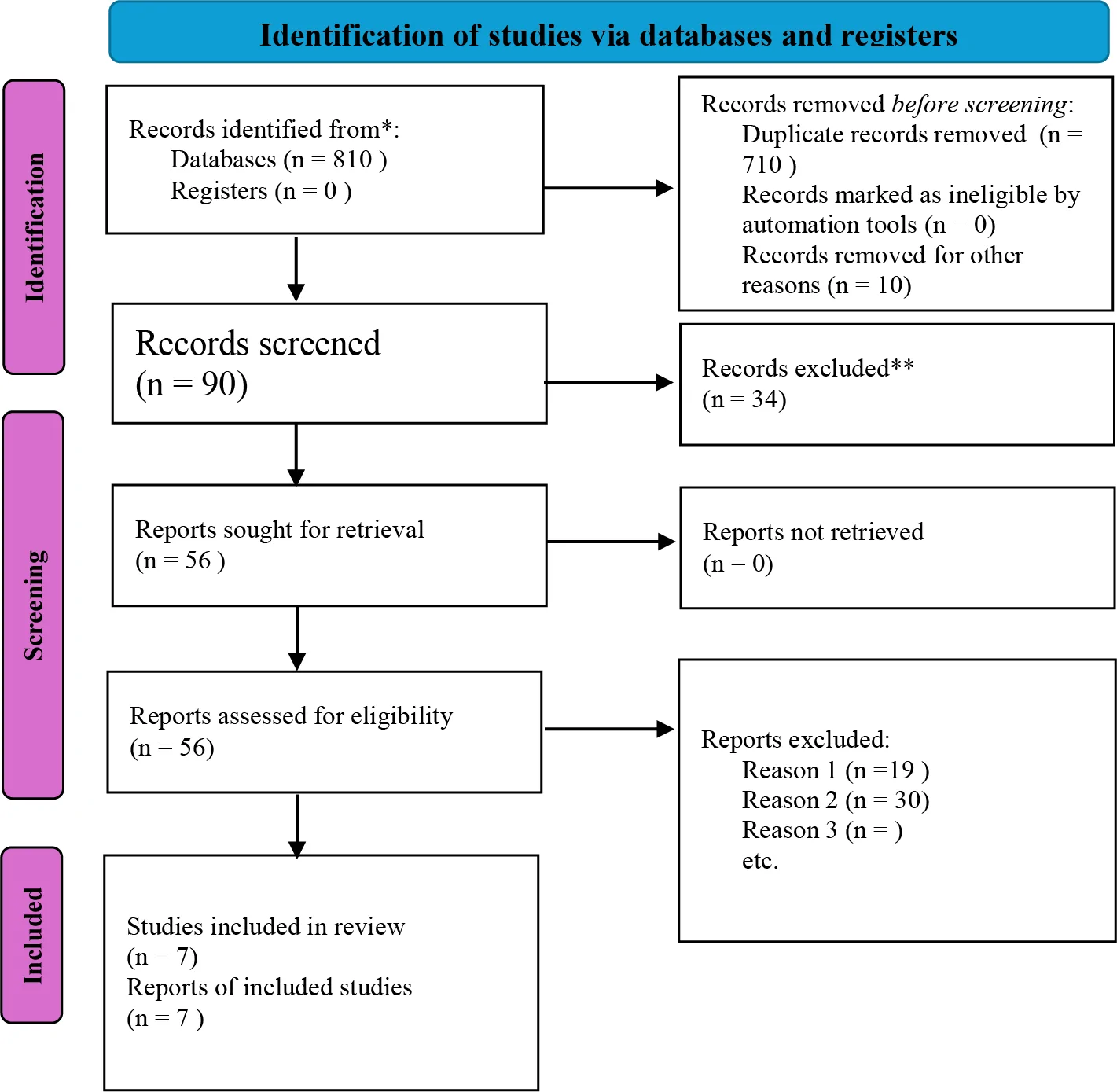
The Prisma Flow diagram of study selection and screening process

Given the rarity of suicide deaths and the limited number of studies that simultaneously restrict samples to children or adolescents, stratify by cognitive ability, and distinguish comorbid ASD+ADHD, it was not feasible to confine this review solely to youth with ASD/ADHD and high IQ. Instead, we prioritized youth and young‑adult findings when available, and drew on adult and mixed‑age cohorts to illuminate mechanisms (e.g., camouflaging, diagnostic overshadowing, comorbidity) that begin in childhood but often become fully apparent only in adulthood and compare outcomes between those autistic and ADHD populations with higher cognitive ability versus those with co-occurring intellectual disability (Fig. 1).

## Results

### The prevalence of suicidal thoughts and behaviors in ASD populations

The recent empirical literature as summarized in Table 1 reports that individuals with ASD have substantially higher rates of STB than the general population across both 12-month and lifetime intervals.

**Table 1:** Summary of empirical studies linking suicidality in youths with ASD

Author (Year)	Study objective	Design and sample	Principal findings
Kirby et al. [12]	Assess suicide incidence in individuals with ASD over two decades	Linkage of four statewide administrative datasets in Utah	Suicide incidence in ASD was 6.4 per 10,000 individuals between 2013 and 2017. Individuals with ASD died by suicide at a younger mean age than non-autistic comparators.
Casten et al. [13]	Examine suicidality in autistic individuals with high cognitive ability (IQ ≥ 120)	Clinical ASD cohort (*N* = 1,074), SPARK cohort (*N* = 16,049), and ABCD cohort (*N* = 11,878)	High cognitive ability was associated with increased suicidal ideation in ASD. Individuals with both ASD and high IQ (twice exceptional) demonstrated the highest risk.
O’Halloran et al. [14]	Review prevalence and risk factors for suicidality in autistic youth	Systematic review and meta-analysis	Identified key risk factors included bullying, trauma exposure, emotional dysregulation, psychiatric comorbidities, and gender dysphoria.
Huntjens et al. [15]	Estimate lifetime prevalence of suicidal ideation and attempts in ASD	Meta analysis of studies published 1990–2022 using PRISMA guidelines	Pooled lifetime prevalence estimates were 37.2% for suicidal ideation and 15.3% for suicide attempts.
Brown et al. [16]	Update evidence on suicide prevalence, risk factors, and interventions in ASD	Systematic review (2018–2024)	Pooled prevalence estimates were 34.2% for suicidal ideation and 24.3% for suicide attempts. Interpersonal stressors and psychiatric comorbidities were key risk factors across the lifespan.
Masi et al. [17]	Examine suicidality in adolescents with high functioning ASD and bipolar disorder	Inpatient clinical study (*N* = 52) comparing ASD, bipolar disorder, and suicidality subgroups	Adolescents with co-occurring ASD, bipolar disorder, and suicidal ideation had higher IQ, greater internalising symptoms, and more lethal suicide attempts than comparison groups.
Hu et al. [18]	Assess the impact of cyberbullying on suicidality in high functioning ASD	Outpatient adolescent cohort (*N* = 219)	Cyberbullying victimisation was associated with increased depression, anxiety, and suicidality among autistic adolescents.

A meta-analysis by Huntjens et al. reported pooled lifetime prevalence estimates of 37.2% for SI and 15.3% for SA among individuals with ASD, exceeding that of the general population [15]. Elevated suicide risk extends beyond formally diagnosed ASD. In an analysis of 372 coroners’ inquest records from two regions in England, Cassidy et al. (2022) identified ASD or clinically significant autistic traits in 10.7% of individuals who died by suicide, a rate approximately eleven times higher than the estimated population prevalence of ASD in the United Kingdom [19]. Importantly, autistic traits within the clinically concerning range were overrepresented among those who died by suicide regardless of prior diagnostic status, underscoring the relevance of dimensional ASD features as indicators of vulnerability (Table 2).

The construct of autistic traits or subclinical autistic traits remains debated, given phenotypic overlap with personality disorders and other psychiatric conditions and concerns regarding diagnostic specificity [16]. Despite this, converging evidence across clinical and nonclinical samples demonstrates a robust association between elevated autistic traits and increased risk of STB. These findings support the clinical relevance of autistic traits as markers of suicide risk even in the absence of full DSM 5 criteria for ASD. This is particularly salient in individuals with higher intellectual ability, who may employ sophisticated compensatory strategies that obscure core social communication difficulties and cognitive rigidity during clinical assessment. In such cases, a provisional and longitudinal diagnostic approach may be adopted in clinical settings. Given the complex presentation of ASD with comorbid ADHD and the emergence of personality structures during adolescence, it needs serial assessments to mitigate diagnostic overshadowing [20]. From a suicide prevention perspective, subclinical autistic traits should therefore be regarded as a meaningful risk signal, while explicitly acknowledging nosological uncertainty and maintaining openness to diagnostic revision. Newell et al. demonstrated that undiagnosed individuals with clinically significant autistic traits are at increased risk of suicide, reinforcing the importance of incorporating dimensional ASD symptoms into suicide prevention and risk assessment frameworks [21].

Gender differences further highlight challenges, as Cassidy et al. (2022) reported that 16% of women with elevated ASD traits had attempted suicide and 45% had formulated a suicide plan, yet only 32% of the overall sample had documented suicidal ideation prior to death. While the families reported being unaware of suicide risk, emphasizing the poor understanding of the lack of conventional warning signs in this population [19].

### Increased risk of suicide and mental health concerns in higher-functioning ASD/ADHD

Emerging evidence suggests that higher intelligence may paradoxically mediate increased risk of STB in neurodivergent populations [13]. Elevated cognitive ability may facilitate compensatory strategies such as social camouflaging and scripting, which can obscure core difficulties, delay diagnosis, and reduce access to appropriate supports [22].

Neurocognitive profiling studies of these ASD youth demonstrate marked variabilities with relative strengths in verbal comprehension alongside significant deficits in processing speed [23]. These profiles contribute to substantial diagnostic delay when compared with ASD cohorts with lower IQ. While these individuals are 14.5 times more likely to reach adulthood without a diagnosis and nine times more likely to remain undiagnosed at time of school entry. Prior to formal ASD diagnosis, many receive alternative psychiatric diagnoses that fail to capture underlying neurodevelopmental vulnerabilities [24].

In a population based study, Casten et al. (2023) found that autistic individuals with IQ above 120 reported the highest rates of suicidal thoughts (12.9%), compared with 2.4% among IQ matched neurotypical controls [13]. These findings suggest that conventional protective factors such as educational attainment, age, or marital status confer less protection against suicidality in autistic populations. Accurate risk assessment is further complicated by parental underreporting and by difficulties among autistic youth in communicating internal states, including impairments in interoception and alexithymia.

Physiological mechanisms may also contribute to elevated risk. Autistic individuals commonly exhibit increased baseline cortisol levels, reflecting chronic activation of the hypothalamic pituitary adrenal axis [25]. Exceptional cognitive ability may amplify this stress response by increasing academic, social, and familial expectations. When these expectations are unmet or unsupported, cumulative stress may further elevate suicide risk in twice exceptional youth.

Mortality studies reinforce these concerns. Individuals with autism have significantly higher all-cause mortality than the general population, with suicide accounting for a disproportionate share of deaths among those without intellectual disability. In a nationwide Swedish cohort, Hirvikoski et al. (2016) reported a 9.4 fold increased risk of death by suicide among autistic individuals without intellectual disability, compared with a 2.4 fold increase among those with co-occurring intellectual disability. Autistic females demonstrated the highest relative risk, with a thirteen fold increase compared with the general population [26]. Although some sex differences may reflect sampling biases favoring cognitively able individuals, more recent data suggest that suicide risk among autistic males and females may be more comparable than previously assumed [27].

Importantly, descriptors such as high functioning or twice exceptional risk minimizing the substantial impairments experienced by these individuals, including executive dysfunction, emotional dysregulation, working memory limitations, and persistent social communication difficulties. These disparities may be especially distressing when they occur in the absence of diagnosis or support [22]. Future research should prioritize stratification by cognitive ability, gender, comorbid psychiatric conditions, and contextual stressors to more accurately characterize suicide risk within autistic populations.

Comorbidity with ADHD further compounds risk. Epidemiological studies estimate that 30 to 50% of individuals with ASD meet criteria for ADHD, with even higher rates observed in clinically referred samples [28]. Joshi and Wilens (2021) reported that up to 75% of psychiatrically referred individuals with ASD met criteria for ADHD, while approximately 15% of those diagnosed with ADHD also met criteria for ASD [29]. This overlap is clinically significant, as both conditions independently increase risk for suicidal thoughts and behaviors, and ADHD related impulsivity may contribute to poorly planned and rapidly enacted suicide attempts [30].

In a large online sample skewed toward cognitively able autistic adults, Moseley et al. found that although only 17.2% reported a formal ADHD diagnosis, 45.2% met threshold criteria for clinically significant ADHD symptoms on standardized measures. The sample was characterized by high educational attainment, with over 70% holding a bachelor’s degree and more than one third holding postgraduate qualifications [31]. These findings highlight substantial under recognition of ADHD among autistic individuals with higher cognitive ability.

Impulsivity associated with ADHD presents unique challenges for suicide prevention, as the decision to attempt suicide may occur within minutes or hours following an acute stressor [32]. Fluctuating suicidal ideation further complicates routine risk assessment in this population [33]. In addition, individuals with co-occurring ASD and ADHD experience elevated exposure to trauma, bullying, social rejection, and perceived burdensomeness, all of which are well established risk factors for suicidality [31].

### Comorbid conditions adding to vulnerabilities

Epidemiological estimates indicate that the risk of death by suicide is approximately 7.5 times higher among autistic individuals than among non-autistic populations [26]. Elevated risk has also been documented in individuals with co-occurring autism spectrum disorder and attention deficit hyperactivity disorder [24], as well as in those with clinically significant autistic traits that do not meet full diagnostic criteria [8]. This excess risk is likely multifactorial and associated with a markedly increased lifetime prevalence of depression, estimated to be approximately four times higher in autistic individuals than in the general population [28]. Additional contributors include higher rates of eating disorders [29], chronic pain conditions [30], exposure to bullying and victimisation [32], and experiences of iatrogenic harm within clinical settings [31].

Exceptional cognitive ability appears to further complicate suicide risk profiles. Evidence suggests that autistic individuals with high intellectual ability (IQ ≥ 120) experience greater internalizing symptoms, lower self-esteem, and higher levels of depressive symptomatology than autistic peers with average cognitive ability. In contrast to neurotypical populations, in which higher intelligence is generally protective against suicide, cognitive ability in autistic individuals may function as a risk modifier. One proposed mechanism is social camouflaging, whereby autistic individuals consciously mask core traits to conform to social norms. While camouflaging may facilitate superficial social integration, it has been consistently associated with increased suicidality, depression, anxiety, and delayed diagnostic recognition [13].

## Discussion

Our prior work has demonstrated that STB in autistic youth are frequently underrecognized because standard suicide risk screening instruments lack sensitivity to autistic phenomenology. We have further shown that specific autistic phenotypes evade gold standard psychometric testing due to comorbidity and diagnostic overshadowing, particularly when attentional, mood, or anxiety symptoms dominate clinical presentations. Treatment gaps compound this vulnerability, as evidence for conventional antidepressant and anxiolytic strategies in ASD remains limited, with frequent atypical responses and heightened sensitivity to adverse effects. This review extends this literature by focusing on a particularly high risk and understudied subgroup: cognitively able youth with co-occurring ASD and ADHD, whose suicide risk remains elevated despite apparent functional success. This phenotype is especially prone to diagnostic misattribution, as overlapping features with personality disorders may obscure the underlying neurodevelopmental etiology. Reviews of ASD without intellectual disability highlight both frequent co-occurrence with cluster A and cluster C personality traits and the risk of conflating neurodevelopmental differences with personality pathology when developmental timing, social cognition, and sensory processing are insufficiently considered. While personality disorders typically emerge in adolescence or early adulthood, ASD reflects a lifelong neurodevelopmental condition with distinct cognitive and interpersonal architecture. Failure to differentiate these trajectories may delay autism informed care and perpetuate risk. By delineating the psychosocial and clinical gaps specific to high functioning ASD with ADHD, this review underscores the need for developmentally anchored, autism informed approaches to suicide risk assessment, differential diagnosis, and intervention. The following section is structured around three critical areas of empirical findings in the results. We first address the counterintuitive suicide risk observed in high functioning youth with autism spectrum disorder, then examine how co-occurring attention deficit hyperactivity disorder contributes to diagnostic delay and compounded vulnerability, and finally outline key mechanisms, clinical gaps, and directions for intervention and systems level change.

### Counterintuitive suicide risk in high functioning autistic youth

The escalating burden of death by suicide among adolescents represents a critical public health concern. Despite increasing empirical attention, suicide risk within neurodevelopmental populations remains incompletely understood. Autism spectrum disorder has historically been conceptualized as a broad and heterogeneous condition, a framing that often obscures meaningful subgroup vulnerabilities. Accumulating epidemiologic evidence now indicates that suicide risk is disproportionately elevated among individuals with ASD without intellectual disability, particularly those with higher cognitive ability. This counterintuitive pattern challenges longstanding assumptions that intellectual functioning or academic success confers protection against suicidality [12, 26, 34].

High functioning autistic youth often evade early identification due to intact academic performance, verbal fluency, and adaptive camouflage [5, 35]. These characteristics contribute to delayed diagnosis and prolonged exposure to neurodevelopmental and epigenetic stressors without appropriate supports. As a result, mental health difficulties frequently emerge through atypical phenomenological pathways that are poorly captured by conventional psychiatric models. Rather than overt behavioral dysregulation, distress in this population may manifest as internalized anxiety, affective instability, rigid or ruminative thinking, and increasing social withdrawal [36]. These presentations are easily misinterpreted or minimized, particularly in youth perceived as gifted, resilient, or high achieving.

Consistent with prior population-based studies, suicidal thoughts and behaviors in ASD appear to be concentrated among cognitively able individuals. Our findings extend this literature by highlighting that this subgroup follows a distinct risk trajectory characterized by late recognition, cumulative social stress, and limited access to autism informed interventions. The paradox of elevated suicide risk among those with high apparent functioning underscores the need to move beyond surface level indicators of wellbeing and toward developmentally and neurobiologically informed models of risk assessment [16, 19].

### How comorbid ADHD compounds diagnostic confusion and risk

When ASD and ADHD co-occur, ADHD is frequently diagnosed earlier, particularly in youth with high cognitive ability and strong academic performance [37]. ADHD related symptoms are often more visible, more familiar to clinicians, and more readily accepted by families and educational systems. Although ADHD treatments may be appropriately optimized, many youths continue to experience persistent difficulties that are not fully explained by attentional or executive dysfunction alone. Developmental differences in social cognition, emotional reciprocity, and adaptive functioning, core features of ASD, are frequently overshadowed by academic strengths and attributed instead to anxiety, mood symptoms, or behavioral challenges.

When ASD remains unrecognized, the interaction between autistic social misattunement and ADHD related impulsivity and emotional dysregulation may substantially amplify vulnerability. High functioning youth often experience chronic peer rejection, bullying, and social exclusion despite academic success, particularly when environmental expectations exceed adaptive capacity. The sustained cognitive and emotional effort required to conform to neurotypical norms through masking or camouflaging can lead to exhaustion, identity strain, and internalized distress. Over time, this cumulative burden may increase reliance on maladaptive coping strategies, including substance use, and contribute to escalating suicidal ideation [21, 38].

Importantly, this risk profile is frequently concealed by surface level indicators of success. Many of these youth are perceived as resilient or gifted and lack conventional warning signs for suicidality. Consequently, parents, teachers, and clinicians are often perplexed by the emergence of acute suicidal crises, which may appear sudden and unanticipated. The absence of validated suicide risk assessment tools for autistic youth further compounds this challenge, as standard measures may fail to capture alexithymia, compensatory masking, and internally experienced distress [39]. Together, these factors render high functioning youth with ASD and co-occurring ADHD particularly vulnerable to being overlooked until crisis occurs.

### What can be done: addressing structural gaps and advancing care

Youth with ASD who exhibit splintered cognitive profiles represent a subgroup with particularly complex clinical presentations shaped by interacting neurodevelopmental and epigenetic pathways. Marked discrepancies between intellectual capacity and adaptive or socioemotional functioning create vulnerability that is often obscured by surface level competence. Epidemiologic data underscore this paradox. For example, a University of Iowa cohort comparing autistic youth of average intelligence demonstrated that those with IQs of 120 or higher were significantly more likely to report STB. Such findings challenge assumptions that cognitive ability is protective and instead suggest that heightened insight, self-comparison, and unmet expectations may amplify risk. However, the phenomenological mechanisms underlying this association remain poorly understood due to limitations in empirical research and inadequately operationalized clinical constructs.

A growing body of evidence implicates camouflaging as a central mechanism linking high functioning ASD to suicidality. Sustained efforts to blend in by concealing autistic traits, scripting social interactions, or suppressing sensory and emotional needs have been consistently associated with anxiety, depression, and suicidal thoughts and behaviors. While camouflaging may facilitate short term social acceptance, it imposes a chronic cognitive and emotional burden that contributes to exhaustion, identity fragmentation, and despair [5,40]. These risks are further magnified by limited recognition from families, teachers, clinicians, and peers, who may misinterpret al.exithymia or emotional reserve as evidence that high achieving youth are coping well. Silence is often mistaken for resilience, delaying intervention until crisis occurs.

Social rejection and bullying, both online and in person, represent potent perpetuating factors in this trajectory [14]. Empirical work demonstrates that repeated experiences of exclusion and victimization intensify internal distress and predict sustained deterioration in mental health among autistic youth [18]. These trauma related responses may be biologically amplified in individuals with dysregulated stress systems, including heightened baseline cortisol and altered hypothalamic pituitary adrenal axis functioning. Over time, cumulative social stress interacts with neurodevelopmental vulnerability to increase risk for suicidal ideation and maladaptive coping.

The double empathy problem offers a critical theoretical lens for understanding these dynamics. Rather than reflecting deficits in empathy, social difficulties in ASD arise from reciprocal misunderstandings between autistic and neurotypical individuals [41]. Autistic people often demonstrate intact or heightened empathic attunement within autistic contexts yet experience persistent misattunement in neurotypical environments. Contemporary research has further challenged deficit-based assumptions by demonstrating that autistic individuals frequently empathize more effectively with other autistic people than with neurotypical peers. This mismatch has significant consequences, contributing to marginalization across social, medical, and educational systems.

These challenges are compounded at the institutional level, giving rise to what has been described as a triple empathy problem. Schools, clinics, workplaces, and legal frameworks are structured around neurotypical norms of communication, emotional expression, and help seeking. As a result, autistic distress is often misunderstood, minimized, or pathologized rather than supported [42]. The expectation that autistic individuals must continuously adapt to environments misaligned with their neurotype contributes to chronic loneliness, fatigue, and hopelessness.

Delayed diagnosis and diagnostic overshadowing further intensify vulnerability, particularly among cognitively able individuals, women, and girls [35]. When ASD is unrecognized, youth frequently receive secondary psychiatric labels without access to identity affirming care. The absence of accommodations related to sensory sensitivity, pacing, predictability, and emotional safety accelerates emotional decline and limits protective buffering. Interventions designed for neurotypical adolescents are often applied without modification, despite evidence that autistic youth differ substantially in affective processing, executive functioning, and sensory responsivity.

Encouragingly, intervention frameworks are beginning to evolve. Autism informed suicide safety planning has shown promise, particularly when co designed with autistic individuals and grounded in empathy-based models. Psychoeducation that redistributes the burden of adaptation away from autistic youth and toward families, educators, and clinicians is increasingly recognized as foundational. Neurodiversity affirming programs, including structured peer-based interventions and autistic led networks, demonstrate emerging benefits in fostering belonging, validation, and social connection, key protective factors against suicidality.

The Stanford Neurodiversity Project REACH (SNP-REACH), a research, education, and advocacy camp for high school students, along with peer-focused social skills initiatives such as the UCLA Program for Excellence in Education and Research in the Sciences (PEERS), exemplify structured, community-based supports that show emerging benefits in reducing social isolation and strengthening protective factors against hopelessness and suicidality [43, 44]. These approaches emphasize environmental fit and relational support rather than individual normalization, aligning intervention goals with autistic lived experience.

Despite these advances, substantial gaps remain. Systematic reviews continue to document alarmingly high rates of suicidal ideation, plans, and attempts among autistic individuals without intellectual disability. Validated suicide screening instruments tailored for autistic populations are scarce, and resources specifically designed for suicide prevention in ASD remain limited. While there is growing evidence that ADHD medication may reduce mortality risk, including suicide, in ADHD populations, comparable evidence in ASD youth is limited. Pharmacologic trials for anxiety and depression in ASD have yielded mixed results, and adverse sensory and neuropsychiatric effects are more common. Future randomized controlled trials must stratify by cognitive ability, comorbid ADHD, age, and sensory profile to meaningfully advance care.

**Table 2:** Suicide risk in youth with autism across neurodevelopmental domains: evidence gaps and implications for prevention

Domain	What is known	Key gaps in evidence	Implications for clinical care and research
Epidemiology of suicide risk	Population based and registry studies consistently show elevated suicidal ideation, attempts, and suicide deaths in individuals with ASD, particularly those without intellectual disability. Risk appears higher when ADHD traits are present.	Few studies specifically examine high functioning ASD youth with co-occurring ADHD as a distinct subgroup. Risk estimates are often diluted by heterogeneous samples.	Targeted epidemiologic studies are needed to quantify risk in this subgroup and identify developmental windows for prevention.
Cognitive ability and risk	Higher cognitive ability in ASD is associated with increased suicidal ideation, challenging assumptions that intelligence is protective.	Mechanisms linking high cognitive ability to suicidality remain poorly defined, especially in adolescents.	Research should examine how self-awareness, expectations, and insight interact with ASD and ADHD traits to elevate risk.
Phenomenology	Distress is often internalized, masked by camouflaging, and difficult to articulate due to alexithymia. ADHD contributes impulsivity and emotional lability.	Limited qualitative and longitudinal data describe phenomenological pathways to suicidality in high functioning ASD youth.	Incorporating lived experience and qualitative methods can clarify pathways that are not captured by standard psychiatric models.
Diagnostic pathways	Delayed diagnosis and diagnostic overshadowing are common, particularly when ADHD is diagnosed first and academic performance is intact.	Few studies examine how diagnostic delay itself contributes to suicide risk trajectories.	Earlier autism informed assessment may represent a modifiable risk factor.
Social stress and trauma	Bullying, peer rejection, and social invalidation are common and associated with worsening mental health and suicidality.	Lack of ASD specific models linking social trauma to suicide risk in youth.	Prevention efforts should address school climate, peer support, and trauma informed care.
Assessment and measurement	Common suicide screening tools are widely used in ASD populations.	Most suicide risk instruments are not validated for autistic youth and do not account for communication differences or alexithymia.	Development and validation of autism informed suicide risk assessment tools is a critical priority.
Psychopharmacology	ADHD medications reduce mortality in general populations; antidepressant and anxiolytic evidence in ASD is limited and mixed.	Few randomized controlled trials stratify by ASD, ADHD, and cognitive ability; sensory sensitivities and atypical responses are underreported.	Future trials should stratify by neurodevelopmental profile and assess tolerability and functional outcomes.
Psychosocial interventions	Neurodiversity affirming approaches, peer support, and adapted safety planning show early promise.	Limited controlled trials evaluate suicide prevention interventions tailored to ASD youth with ADHD.	Intervention development should focus on reducing camouflaging demands and enhancing belonging.
Systems of care	Many youth access mental health services prior to suicide attempts.	Services are often designed for neurotypical adolescents and lack autism informed frameworks.	System level changes in schools and healthcare are needed to reduce missed risk and improve engagement.

## Conclusion

ASD with ADHD is associated with a markedly increased risk of death by suicide, particularly among cognitively able individuals whose subjective experiences are often obscured by apparent functional success. Diagnostic delay, camouflaging, social exclusion, and the limited sensitivity of conventional assessment tools contribute to underrecognition of risk and inadequate intervention. These findings highlight the need for early identification, informed suicide risk assessment, and environments that reduce sensory and social burden. Shifting toward developmentally grounded, neuroaffirming models of care is essential to mitigating preventable suicide risk in this population.

## Limitations

This review is constrained by several important limitations in the existing literature. First, empirical studies examining STB in ASD frequently aggregate heterogeneous populations, limiting the ability to draw conclusions specific to high functioning youth with co-occurring ADHD. Many studies exclude individuals with higher cognitive ability from intensive clinical samples or fail to stratify findings by intelligence, adaptive functioning, or comorbid neurodevelopmental conditions. As a result, risk estimates for this subgroup are likely underestimated. Second, variability in diagnostic practices complicates interpretation. Delayed diagnosis, diagnostic overshadowing, and inconsistent recognition of ADHD in ASD contribute to misclassification and reduce the reliability of reported associations. Many studies rely on parent report or clinical records rather than standardized assessments, and few adequately capture camouflaging behaviors or internalized distress, which are central to the phenomenology of suicidality in this population. Third, measurement limitations remain substantial. Most suicide risk screening tools and symptom rating scales have not been validated for autistic youth, nor do they account for differences in communication style, alexithymia, or literal interpretation of language. This raises concerns about both false negatives and false positives and limits cross study comparability. Fourth, the therapeutic trials in ASD are few, often underpowered, and rarely stratified by cognitive ability or co-occurring ADHD. Adverse effects, sensory sensitivities, and atypical medication responses are inconsistently reported, further limiting clinical generalizability.

Finally, much of the available evidence is cross sectional, restricting causal inference. Longitudinal data examining developmental trajectories from early adolescence through young adulthood are scarce, particularly for youth who appear high functioning but experience significant subjective distress. These gaps constrain our ability to identify early markers of risk and windows for prevention.

### Future directions

Future research must prioritize stratified and developmentally informed approaches to better characterize suicide risk in high functioning ASD youth with co-occurring ADHD. Large scale longitudinal studies are needed to examine how cognitive ability, executive functioning, emotional regulation, and social experiences interact over time to shape trajectories toward suicidal ideation and behavior. Such studies should explicitly include youth who camouflage and may be overlooked in traditional clinical pathways. Development and validation of autism informed suicide risk assessment tools is a critical priority. Measures must account for alexithymia, atypical emotional expression, and neurodivergent communication styles, and should be tested specifically in youth with ASD and ADHD. Incorporating qualitative methods and lived experience perspectives may enhance construct validity and clinical utility. Intervention research should move beyond symptom reduction toward mechanisms relevant to this population. Trials evaluating peer-based interventions, reduction of camouflaging, environmental accommodations, and neuroinclusive school practices are urgently needed. Suicide safety planning should be adapted for autistic youth and co designed with individuals with lived experience, emphasizing clarity, predictability, and sensory considerations. Psychopharmacologic research would benefit from stratification by neurodevelopmental profile, cognitive ability, and age. Randomized controlled trials should examine not only efficacy but tolerability, sensory sensitivity, and functional outcomes in ASD youth with ADHD. Comparative effectiveness studies evaluating combined behavioral and medication approaches may better reflect real world care. Future work should evaluate models of care that integrate educational, clinical, and family supports and that shift responsibility for adaptation away from autistic youth. Training educators and clinicians in neurodevelopmentally informed and empathy-based frameworks may reduce diagnostic delay, improve engagement, and ultimately mitigate suicide risk.

## Data Availability

No datasets were generated or analysed during the current study.

## Abbreviations

ADHD: Attention deficit hyperactivity disorder
ASD: Autism spectrum disorder
DSM: 5–diagnostic and statistical manual of mental disorders, fifth edition
HPA: Hypothalamic pituitary adrenal
IQ: Intelligence quotient
SA: Suicide attempts
SI: Suicidal ideation
STB: Suicidal thoughts and behaviors
SBQ: ASC–suicidal behaviors questionnaire for autism spectrum conditions (SBQ–ASC)
SIDAS: M–suicidal ideation attributes
ASQ: Ask suicide screening questions
